# Selenoprotein P-1 (SEPP1) as an Early Biomarker of Myocardial Injury in Patients Undergoing Cardiopulmonary Bypass

**DOI:** 10.3390/jcm13102943

**Published:** 2024-05-16

**Authors:** Giuseppe Filiberto Serraino, Davide Bolignano, Federica Jiritano, Giuseppe Coppolino, Désirée Napolitano, Mariateresa Zicarelli, Patrizia Pizzini, Sebastiano Cutrupi, Alessandra Testa, Belinda Spoto, Michele Andreucci, Pasquale Mastroroberto, Raffaele Serra

**Affiliations:** 1 Department of Experimental and Clinical Medicine, University “Magna Graecia” of Catanzaro, 88100 Catanzaro, Italy; serraino@unicz.it (G.F.S.); jiritano@unicz.it (F.J.); desiree.napolitano@unicz.it (D.N.); mastroroberto@unicz.it (P.M.); 2 Department of Medical and Surgical Sciences, University “Magna Graecia” of Catanzaro, 88100 Catanzaro, Italy; dbolignano@unicz.it (D.B.); mteresa.zicarelli@gmail.com (M.Z.); 3 Department of Health Sciences, University “Magna Graecia” of Catanzaro, 88100 Catanzaro, Italy; gcoppolino@unicz.it (G.C.); andreucci@unicz.it (M.A.); 4 Italian National Council of Research (CNR)—Institute of Clinical Physiology, 89100 Reggio Calabria, Italy; ppizzini@ifc.cnr.it (P.P.); scutrupi@ifc.cnr.it (S.C.); atesta@ifc.cnr.it (A.T.); bspoto@ifc.cnr.it (B.S.); 5 Interuniversity Center of Phlebolymphology (CIFL), International Research and Educational Program in Clinical and Experimental Biotechnology, Department of Medical and Surgical Sciences, University Magna Graecia of Catanzaro, Viale Europa, 88100 Catanzaro, Italy

**Keywords:** cardiac surgery, biomarker, SEEP1, selenoprotein 1, cardiopulmonary bypass, myocardial injury, coronary artery bypass graft, postoperative myocardial injury, cardiovascular biomarkers

## Abstract

**Background**: Biomarkers development for prognostication or prediction of perioperative myocardial disease is critical for the evolution of treatment options in patients undergoing cardiac surgery. The aim of our prospective monocentric study was to investigate the role of selenoprotein 1 (SEEP 1) as a potential biomarker for assessing the risk of myocardial injury after cardiac surgery. **Methods**: Circulating SEPP1 was measured in the blood of 45 patients before surgery and at 4 h, 8 h and 12 h after CPB by enzyme-linked immunosorbent assay (ELISA); (3) **Results**: circulating SEPP-1 levels measured 4 h after surgery were strongly correlated with CK-MB levels measured at 48 h (R = 0.598, *p* < 0.0001) and at 72 h (R = 0.308, *p* = 0.05). Close correlations were also found between 4 h SEPP-1 and Hs-c troponin values measured at 24 h (R = 0.532, *p* < 0.0001), 48 h (R = 0.348, *p* = 0.01) and 72 h (R = 0.377, *p* = 0.02), as well as with cardiopulmonary bypass (CPB) (R = 0.389, *p* = 0.008) and cross-clamp time (R = 0.374, *p* = 0.001); (4) **Conclusions**: Early SEPP1 measurement after CPB may hold great potential for identifying cardiac surgery patients at risk of developing perioperative myocardial injury.

## 1. Introduction

One of the main causes of death and disability in the world is cardiovascular disease. Heart surgery has been shown to have positive prognostic and clinical effects and normally requires cardiopulmonary bypass to be performed [1]. Regretfully, an ischemic event resulting from a graft failure, an abrupt coronary event affecting the native coronary arteries, or insufficient cardioprotection during cardiac surgery might cause a perioperative myocardial infarction.

After myocardial injury, cardiac troponins are generated and removed from the blood slowly, which makes it more difficult to diagnose recurrent acute MI early. As a result, diagnosing perioperative MI in the 48 h following surgery can be extremely difficult [2,3]. Therefore, the diagnostic and therapeutic approach may benefit from an early MI biomarker in patients receiving CPB.

Thanks to developments in molecular biology and biochemistry, many tissue peptides and proteins have been identified in the past ten years that may have a role in the AMI diagnosis. Making thorough risk assessments of patients with myocardial injury as soon as possible and preventing any unfavorable outcomes are the primary goals of the hunt for novel markers.

According to a pilot research study we recently published, early assessment of selenium P-1 (SEPP1) following cardiopulmonary bypass grafting (CPB) may be very beneficial for enhancing renal risk stratification in patients undergoing heart surgery [4]. Although SEPP 1’s existence was initially documented over thirty years ago, the last 10 years have seen many advancements in our understanding of its functions. Mice lacking or altered in SEPP1 have led to the greatest advancements. 

As a selenium transporter, SEPP1 is a protein that is mostly secreted from the liver. It provides tissues and organs with this trace mineral, which activates certain glutathione peroxidase selenoenzymes (GPxs) [5]. Furthermore, SEPP1 appears to have direct extracellular ROS-detoxifying abilities [6,7]. In patients receiving CPB, elevated SEPP1 levels are indicative of myocardial hypoxia and may be predictive of unfavorable outcomes related to the heart, including mortality, arrhythmia, and cerebral ischemia. Based on this, we conducted a subgroup analysis of our observational, pilot, hypothesis-driven study and tested SEPP1’s potential function as a predictive biomarker of MI in the context of cardiac surgery [4]. 

## 2. Materials and Methods

### 2.1. Study Design and Patient Enrollment

This pilot observational study evaluated 198 patients who were consecutively referred to the University Hospital of Catanzaro (Italy) for cardiac surgery between July 2020 and February 2021. Emergency cardiac surgery, being unable to give informed consent, being less than 18 years old, acute concurrent infections, long-term immunosuppressive medication, or having a seriously compromised heart were among the exclusion criteria. The local university Institutional Review Board approved the study, and each participant gave written informed consent.

### 2.2. Clinical Data and SEPP1 Measurement

Before surgery and at four, eight and twelve hours afterward serum samples were collected for SEPP1 determination. After the samples were centrifuged at 1227× *g* for 15 min at 4 °C, aliquots of the serum samples were kept at −80 °C until they were all thawed for batch analysis. The same laboratory (CNR-Institute of Clinical Physiology, Reggio Calabria, Italy) conducted all SEPP1 measurements using a commercially available ELISA kit (Human Selenoprotein P1 ELISA kit, Cloud-Clone Corp.). 

### 2.3. Statistical Analysis

#### Statistics

SPSS software (version 24.0.0.0; IBM Corporation, Armonk, NY, US) and MedCalc statistical software (version 14.8.1; MedCalc Software bvba, MedCalc Software Ltd, Ostend, Belgium) were used to conduct the statistical analysis reporting data as mean ± SD, median [IQ range], or frequency %. 

The statistical variance of SEPP1 and cardiac biomarkers over the designated time points (*p* for trend) was analyzed using one-way ANOVA. Quadratic and linear assumptions were tested.

The clinical correlates of SEPP1 assessed at the designated time-points were found using the Pearson (R) correlation coefficient. All variables exhibiting a skewed distribution were log-transformed in order to more closely resemble normal distributions prior to correlation testing. Every outcome was deemed noteworthy if the *p*-value was less than 0.05.

## 3. Results

### 3.1. Study Population Characteristics

Forty-five consecutive patients undergoing elective major heart surgery with CPB made up the final research population (the main reason for withdrawing the patient was the presence of impaired renal function and/or non-elective surgery) (Table 1). Isolated CABG was the most common surgical procedure (71.1%). The mean age was 65.6 ± 8 years. Patients had a median BMI of 27.8 [IQR 25.9–30.9] and were primarily male (77.8%). Diabetes was quite common (60%) with a median time since onset of 7.5 years [IQR 1–12]. Nearly every person (95.6%) had a history of cardiovascular or cerebrovascular illness in the past. With mean serum creatinine levels of 0.90 ± 0.19 mg/dL and a mean estimated GFR (CKD-EPI) of 91.8 ± 14.3 mL/min/1.73 m^2^, all patients demonstrated normal renal function. Overall, the left atrial volume was 42.3 ± 8.27 mL/m^2^, normal to mildly aberrant, and the ejection fraction was intact (median 50%, IQR 45–55). 

### 3.2. Trends in Cardiac Biomarkers and SEPP1 Levels after Cardiac Surgery

Surgery was successful and well tolerated in all patients with no major complications or adverse events reported. The median cross-clamp time was 72 [IQR 56–104.2] minutes while the median CPB duration was 105 [IQR 91–137] minutes.

A rise in CK-MB was documented 24 h after surgery (25.4 [17.7–61.1] vs. 1.6 [1.3–2.4] UI/L at baseline), with levels returning close to pre-surgery values at 72 h (3.2 [1.9–5.5] UI/L; *p* for trend < 0.0001). A similar time-pattern was described for myoglobin (24 h peak: 501.1 [271–792.5] vs. baseline values: 29 [22.5–45.7] ng/mL; 72 h levels: 114.5 [69.7–207] ng/mL; *p* for trend <0.0001). Conversely, Hs-c troponin showed a substantial increase 24 h after surgery (488 [247.3–1089] vs. baseline values 16 [9.3–26.6] ng/L), with levels remaining steadily high up to 72 h (356.5 [182.1–953.9] ng/L; *p* for trend = 0.01).

**Figure 1:**
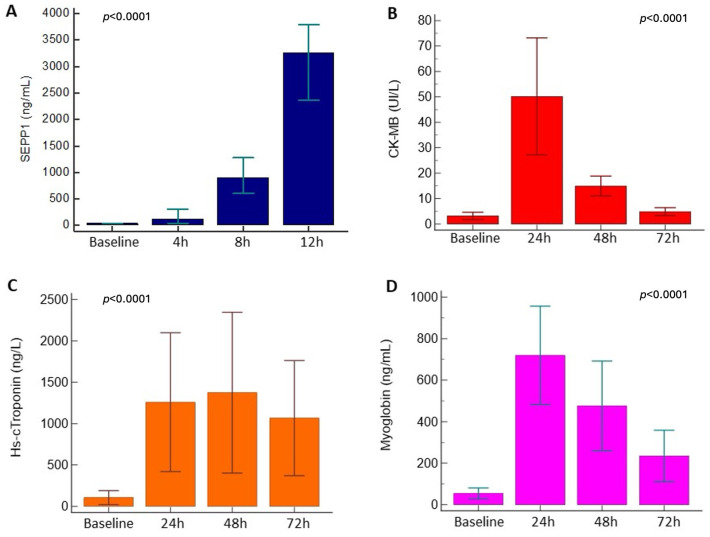
Temporal trend in SEPP1 (**A**), CK-MB (**B**), Hs-c troponin (**C**) and myoglobin (**D**) in the study cohort and statistical significance (*p* for trend).

From baseline to 12 h following the surgery, SEPP1 showed an increasing trend in all patients (69 [IQR 39–85] to 3263 [IQR 1886.2–5042.7] ng/mL; *p* for trend <0.0001). In particular, SEPP1 levels increased by a median of 1.66 [IQR 1–5.01] fold 4 h after surgery. They also increased by 5.35 [IQR 2.55–15.2] fold from 4 to 8 h and by 3.42 [IQR 2.27–5.34] fold from 8 to 12 h (all *p* <0.0001) after surgery. The pre-surgery delta increase from baseline to 12 h after surgery was as high as 53.1 [IQR 33.4–94.6] fold (*p* < 0.0001). The temporal trend of SEPP1 and other cardiac biomarkers in the research population is summarized in Figure 1.

### 3.3. Correlates of SEPP1 Measured at Different Time Points

**Figure 2:**
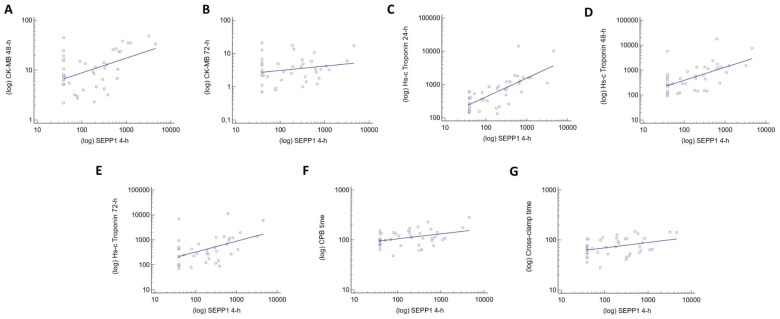
Clinical correlations between SEPP1 measured at 4 h after CPB and CK-MB measured at 48 h (**A**), CK-MB measured at 72 h (**B**), Hs-c troponin measured at 24 h (**C**), Hs-c troponin measured at 48 h (**D**), Hs-c troponin measured at 72 h (**E**), CPB time (**F**) and cross-clamp time (**G**).

In Pearson analyses, circulating SEPP-1 levels measured 4 h after surgery were strongly correlated with those of CK-MB measured at 48 h (R = 0.598, *p* < 0.0001) and 72 h (R = 0.308, *p* = 0.05). Close correlations were also found between 4 h SEPP-1 and Hs-c troponin values measured at 24 h (R = 0.532, *p* < 0.0001), 48 h (R = 0.348, *p* = 0.01) and 72 h (R = 0.377, *p* = 0.02), as well as with CPB (R = 0.389, *p* = 0.008) and cross-clamp time (R = 0.374, *p* = 0.001). SEPP-1 levels measured 8 h after surgery were directly associated with CK-MB values at 48 h (R = 0.460, *p* = 0.01), Hs-c troponin at 24 h (R = 0.395, *p* = 0.007) and CPB time (R = 0.310, *p* = 0.03). Conversely, SEPP1 values measured at baseline and 12 h after surgery did not show significant correlations with any of the variables recorded in this population. Figure 2, and Table 2 summarize the clinical correlates of SEPP1.

## 4. Discussion

Cardiovascular disease is the most common disease all over the world and surgical treatment still represents the best option for many patients with severe conditions not suitable for medical or percutaneous interventions. Most of the time, cardiac operations are performed by using an extracorporeal circuit to oxygenate and pump blood while the heart is not beating due to cardioplegia injection.

Unfortunately, CPB foreign circuits, surgical trauma, graft failure or inadequate myocardial protection could lead to myocardial injury of various severity degrees. Immediate recognition of these conditions helps clinicians and subsequentially patients to receive proper treatment before irreversible damage occurs. Ultrasensitive troponin, myoglobin and CK-MB are well validated biomarkers of myocardial disease, regardless of the type of MI, but sometimes not helpful in this context. Cardiac troponin (cTn) acts on myocardial contraction by regulating the calcium-dependent interaction of actin and myosin; CK is an enzyme that catalyses the reversible transformation of creatine and ATP to creatine phosphate and ADP; and myoglobin is an iron- and oxygen-binding protein abundantly present in the heart and skeletal muscle. All of them are released in the blood during myocardial injury with different sensitivities and specificities, and they are released at different times after cardiac injury [2]. For this reason, myoglobin and CK-MB isoenzymes are no longer used in emergency department settings, as recommended by current guidelines. Nowadays, the combination of clinical signs, ECG, and cardiac biomarkers (troponins) lead to a secure diagnosis of myocardial infarction in the emergency setting.

Serum levels of cTn are modest in healthy individuals, but they rise to a measurable level in cases of myocyte injury because they are released from the contractile apparatus late and from the cytosolic pool promptly. Consequently, following acute myocardial injury, blood levels rise at 2–4 h and peak at 24 h. For two to three weeks, the blood has high troponin levels. Thanks to advances in single-molecule counting technology and high-precision troponin analysis, modern troponin tests are now able to identify even extremely low amounts of troponin [2]. 

In the cardiac surgical context, where the diagnosis of MI should be made during or immediately after the operation and where we expect iatrogenic myocyte damage, a new *early* biomarker could help identify a population at risk for perioperative MI based on its blood level rise during or immediately after the operation. Results from our previous pilot study pointed at SEPP1 as a novel candidate biomarker for early AKI risk stratification in patients undergoing cardiac surgery [4]. A crucial trace element that influences redox signaling and the production of selenoproteins is selenium (Se). In addition to a range of dietary sources of selenium, there are variations related to sex, age, disease, and genotype that affect selenium metabolism and regulation. Se-related proteins can be divided into two main categories: the less well-defined Se-binding proteins and the selenoproteins, which have one or more genetically encoded selenocysteine residues in their core sequence. The most thoroughly characterized protein in the latter category is called selenium-binding protein 1. In electrophoretic studies, selenium-binding protein 1 migrates as a 56 kD band and is expressed in the majority of human and rodent tissues [8]. As reported by other authors [7], we found in our study a remarkable increase in SEPP1 levels after CPB in the whole study cohort, with circulating values peaking up to 53-fold 12 h after surgery, probably representing a compensatory response to the systemic oxidative stress induced by the extracorporeal procedure [9,10]. 

A new finding in this study is the identification of correlations between troponin I, CK MB and SEPP1 over time. SEPP1 displayed an earlier (4–8 h) and more prominent increase compared to the other biomarkers, while such levels reached comparable values at later measurements. In an attempt to identify MI in symptomatic patients, many researchers have investigated the diagnostic efficiency of oxidant biomarkers levels alone or in combination with creatine kinase (CK)-MB and troponin I or myoglobin [11]

Increased SEPP1 levels in patients receiving CPB reflect myocardial hypoxia and may predict unfavorable cardiovascular events such as mortality, bradycardia, or cerebral ischemia. SEPP1 appears to have direct ROS-detoxifying powers at the extracellular level [6]. In actuality, it is believed that oxidative stress is crucial for heart remodeling and is in charge of the development and spread of cardiovascular illness [11]. Current studies on the etiology and development of this condition concentrate on how oxidative stress is caused by the breakdown of normal homeostatic processes. It happens when the body’s antioxidant defense systems and reactive oxygen or nitrogen species (ROS/RNS) are out of balance [12]. Overproduction of ROS has the potential to upset the balance by surpassing the antioxidant capability of cells [13]. This could then lead to DNA mutagenesis, lipid and protein peroxidation, and other potentially harmful outcomes. [14]. Apart from causing direct harm to cells, oxidative stress also triggers mitochondrial malfunction and encourages the production of free radicals, which further intensifies the illness load [15,16]. Selenium and its binding proteins seem to be directly involved in this process, highlighting the potential role of these biomarkers in the cardiac field.

Indeed, after myocardial infarction, serum has been found to include the intracellular protein known as selenium-binding protein 1 (SELENBP1). Heart surgery and hypoxia have an impact on selenium levels and selenoprotein expression. Circulating SELENBP1 concentrations in patients undergoing cardioplegia and cardiopulmonary bypass during cardiac surgery were examined by Khun-Heid et al. [17]. Serum SELENBP1 concentrations were observed to have significantly increased during the intervention and to positively correlate with the length of ischemia (ρ = 0.6, *p* < 0.0001). Predictive markers of patients at risk of unfavorable outcomes (death, bradycardia, or cerebral ischemia) included elevated serum SEEP1 concentrations at 1 h after admission to the intensive care unit (post-surgery) and circulating SELENBP1 during the intervention (2 min after reperfusion or 15 min after weaning from the CPB). Regretfully, the specific mechanism by which pro- and anti-oxidative variables contribute to the difficulties of cardiac illnesses remains incompletely understood. 

Furthermore, Khun et al. discovered a correlation between CK-MB and myocardial damage in their investigation. They discovered that the serum concentration of SEEP1 increased intraoperatively (2 min after reperfusion or 15 min after weaning from the CPB), and that it correlated positively with the CK-MB values measured after the intervention. This is in contrast to previous studies that found circulating concentrations of SEEP1 were unrelated to many routine laboratory markers in patients attending the emergency ward for chest pain, like troponin T, creatinine, the heart-specific isoform of creatine kinase (CK-MB), the liver enzymes, white blood cell count (WBC), potassium, or others. This finding suggests that an early event, a rise in serum SEEP1 concentration, preceded an increase in CK-MB concentration, a marker for myocardial injury. In our analysis, we tested all myocardial markers routinely used and previously described, identifying a direct relationship between SEEP1 and CK-MB, troponin C and myoglobin. Given this, SEPP1 concentration application would be worthy of further investigation as it may aid in better identification of perioperative myocardial illness following heart surgery—a condition that is regrettably still rather common and difficult to anticipate. 

Our study’s primary drawback is its small sample size, which made it difficult to conduct a more thorough analysis and might have underpowered the investigation when compared to the secondary composite endpoint. It is important to take into account the screening biomarkers’ limitations. Comparable to a biological analyte, the concentrations of biomarkers are widely distributed. Therefore, concentrations in individuals with and without disease overlap significantly, even though the underlying distribution varies according to disease status [7]. At the same time, we should be aware that an ideal biomarker for myocardial injury does not exist.

Apart from the reliability, fast use, and low costs of the biomarkers routinely used in clinical settings, we still do not know about the severity or the reversibility of the myocardial injury by measuring them. This has great impact on the patient and on the health system’s costs. On the other hand, our study cohort was relatively homogeneous in terms of comorbidities and types of surgery and did not include patients with acute myocardial infarction. Given the observational nature of this study, the presence of selection bias and significant residual confounding cannot thus be fully ruled out. Finally, SEPP1 was measured up to 12 h after surgery; although previous evidence suggests that SEPP1 levels revert to normal by 24 h after CPB; an extended observation would have been more helpful in characterizing the dynamic relationship between SEPP1 and myocardial injury.

## 5. Conclusions

New biomarkers of myocardial injury are required in the cardiac surgical context. Patients undergoing cardiac surgery who are at risk of experiencing perioperative myocardial damage may benefit greatly from early SEPP1 assessment following CPB. The results of this study, however, should only be regarded as preliminary, and they should be expanded upon in larger and more diverse populations. 

## Figures and Tables

**Table 1:** Baseline clinical and laboratory characteristics of the study cohort.

Patients’ characteristics	
Age (yrs)	65.6 ± 8
Gender (% Male)	77.8
BMI (kg/m)^2^	27.8 (25.9–30.9)
Smoking (%)	35.5
CV disease (%)	95.6
Hypertension (%)	73.3
Diabetes (%)	60
Diabetes vintage (yrs)	7.5 (1–12)
NYHA class (%1/2/3)	15.6/64.4/20
Ejection fraction (%)	50 (45–55)
Left atrial volume (mL/m^2^)	42.3 ± 8.27
Creatinine (mg/dL)	0.90 ± 0.19
eGFR CKD-EPI (mL/min/m^2^)	91.8 ± 14.3
Hemoglobin (g/dL)	12.8 ± 1.5
Hematocrit (%)	38.7 ± 4.7
Total cholesterol (mg/dL)	142.7 ± 43.1
LDL cholesterol (mg/dL)	80 (62.2–108.7)
Triglycerides (mg/dL)	102 (86–131)
Pre-operative medications	
ACEi/ARBs (%)	91.1
Diuretics (%)	44.4
Beta-blockers (%)	82.2
Calcium channel blockers (%)	15.5
Statins (%)	84.4
Platelet inhibitors (%)	28.9
Surgical characteristics	
Type of surgery	
CABG only (%)	71.1
CABG plus valve (%)	15.6
Valve only (%)	11.1
Other (%)	2.2
Pre-operative SBP (mmHg)	130.1 ± 15.7
Pre-operative DBP (mmHg)	74.6 ± 10.9
STS mortality score	1.13 (0.70–2.40)
Cross-clamp time (min)	72 (56–104.2)
CPB time (min)	105 (91–137)
Biomarkers	
CK-MB (UI/L)	1.6 (1.3–2.4)
Hs-cTN (ng/L)	16 (9.3–26.6)
Myoglobin (ng/mL)	29 (22.5–45.7)
SEPP1 (ng/mL)	69 (39–85)

Footnotes: BMI, body mass index; CV, cardiovascular; NYHA, New York Health Association; eGFR, estimated glomerular filtration rate; LDL, low density lipoprotein; ACEi, angiotensin-converting enzyme inhibitors; ARBs, angiotensin receptor blockers; CABG, coronary artery bypass graft; SBP, systolic blood pressure; DBP, diastolic blood pressure; STS, short term score; CPB, cardiopulmonary bypass; CK-MB, creatine kinase MB; Hs-CTN: high-sensitivity c-troponin; SEPP1: selenoprotein-P1.

**Table 2:** Clinical correlates (Pearson coefficient) of SEPP1 measured at 4 h and 8 h, respectively.

*SEPP1 4* h	R	*p*
CK-MB 48 h	0.598	<0.0001
CK-MB 72 h	0.308	0.05
Hs-c Troponin 24 h	0.532	<0.0001
Hs-c Troponin 48 h	0.348	0.01
Hs-c Troponin 72 h	0.377	0.02
CPB time	0.389	0.008
Cross-clamp time	0.374	0.001
*SEPP1 8* h	R	*p*
CK-MB 48 h	0.460	0.01
Hs-c Troponin 24 h	0.395	0.007
CPB time	0.310	0.03

Footnotes: SEPP1: selenoprotein-P1; CK-MB, creatine kinase MB; Hs-CTN: high-sensitivity c-troponin; CPB, cardiopulmonary bypass.

## Data Availability

All research data are available and could be shared by emailing the corresponding author.
